# Development and Function of the Cardiac Conduction System in Health and Disease

**DOI:** 10.3390/jcdd4020007

**Published:** 2017-06-07

**Authors:** David S. Park, Glenn I. Fishman

**Affiliations:** Leon H. Charney Division of Cardiology, New York University School of Medicine, New York, NY 10016, USA; david.park@nyumc.org

**Keywords:** cardiac conduction system development, ventricular conduction system, gene regulatory networks

## Abstract

The generation and propagation of the cardiac impulse is the central function of the cardiac conduction system (CCS). Impulse initiation occurs in nodal tissues that have high levels of automaticity, but slow conduction properties. Rapid impulse propagation is a feature of the ventricular conduction system, which is essential for synchronized contraction of the ventricular chambers. When functioning properly, the CCS produces ~2.4 billion heartbeats during a human lifetime and orchestrates the flow of cardiac impulses, designed to maximize cardiac output. Abnormal impulse initiation or propagation can result in brady- and tachy-arrhythmias, producing an array of symptoms, including syncope, heart failure or sudden cardiac death. Underlying the functional diversity of the CCS are gene regulatory networks that direct cell fate towards a nodal or a fast conduction gene program. In this review, we will discuss our current understanding of the transcriptional networks that dictate the components of the CCS, the growth factor-dependent signaling pathways that orchestrate some of these transcriptional hierarchies and the effect of aberrant transcription factor expression on mammalian conduction disease.

## 1. Introduction

**Figure 1:**
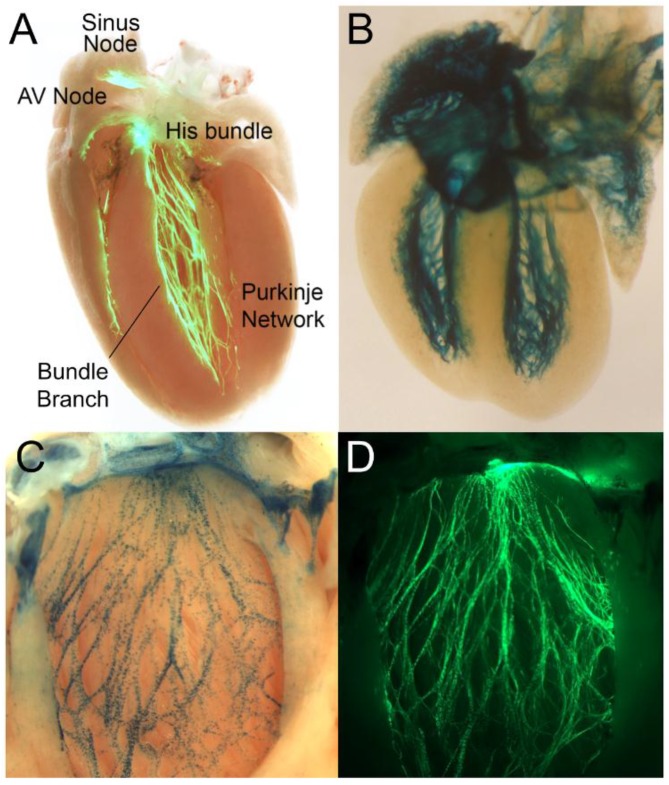
Cardiac conduction system (CCS) reporter mice. (**A**) Contactin2-eGFP demonstrating CCS components (Reproduced with permission from [1]); (**B**) cardiac conduction system reporter-LacZ in an embryonic day 17.5 heart (Reproduced with permission from [2]); (**C**,**D**) co-expression of Etv1-nuclear LacZ (**C**) in a Contactin2-EGFP; (**D**) background delineating the left ventricular conduction system. AV, atrioventricular.

The CCS is functionally divided into the impulse generating, but slowly conducting nodal cells and the rapidly-conducting ventricular conduction system (VCS); as visualized using various conduction system reporter mice (Figure 1). The dominant pacemaker is the sinus node (SN) located at the junction between the superior vena cava and the right atrium (RA). Upon exiting the SN, impulses travel rapidly through the atrial myocardium, ensuring synchronous contraction of the atrial chambers. The cardiac impulse then slows in the atrioventricular node (AVN), which is the last point of communication between the atria and ventricles, providing adequate time for ventricular filling. The impulse accelerates again as it enters the penetrating His bundle, which traverses the central fibrous body crossing the annulus fibrosus that electrically isolates atria from ventricles, and then, rapidly disseminates throughout the ventricular myocardium using the VCS, also referred to as the His-Purkinje system (HPS). The VCS includes the His bundle, left and right bundle branches and distal Purkinje fiber network. The HPS allows for: (1) apex-to-basal ventricular contraction; (2) left and right ventricular synchrony; and (3) intraventricular synchrony. This coordinated electrical activity is highly conserved in all mammalian species and is essential to maintain optimal stroke volume.

**Figure 2:**
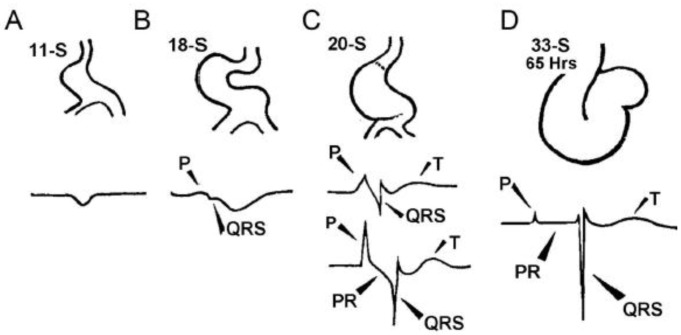
Schematic of chicken heart development and corresponding electrocardiograms at somite stages shown. (**A**) Somite Stage 11 (11-S); (**B**) 18-S; (**C**) 20-S; and (**D**) 33-S (Reproduced with permission from [3]).

The formation of the CCS occurs simultaneously with cardiac development [3] (Figure 2). Heart formation begins as a linear tube that maintains circulation via peristaltic contraction. At this stage, the linear heart tube is composed of primary heart field myocardium and conducts impulses slowly. Electrocardiographic (ECG) recordings at this stage in developing chicks show a sinusoidal waveform. At the onset of looping morphogenesis, the ECG morphology changes to show distinct P waves (represents atrial activation on ECG) and QRS waves (represents HPS-dependent ventricular activation on ECG) that are separated by a PQ or PR interval (represents cumulative measure of atrial, AVN and HPS-dependent ventricular activation time on ECG). At the same time, the atrial and ventricular electrocardiogram signals acquire high frequency waveforms, indicative of rapid conduction. Rapid conduction is a hallmark feature of cardiac chamber formation, where pectinated myocardium of the atria and trabeculated myocardium in the ventricles adopt a fast conduction phenotype. Subendocardial trabeculated cardiomyocytes in the ventricles undergo further specification to form the highly specialized VCS, whereas in the atria, the pectinated atrial myocardium maintains the fast conduction phenotype without further specification. The atrioventricular (AV) junction forms a constriction called the AV canal (AVC) that maintains the slow conduction phenotype of the linear heart tube, serving as the nascent AVN. Eventually, epicardial cells ingress into the ventricular aspect of AV junctional myocardium giving rise to the annulus fibrosus, except in the dorsal aspect, where the AVN maintains electrical continuity with the penetrating His bundle. The remaining AV junctional myocardium remains into adulthood as the AV ring bundles [4].

The gene regulatory networks that govern CCS development are tightly orchestrated in a tissue-specific and time-dependent manner, producing distinct nodal and fast conduction phenotypes. The nodes display high levels of automaticity driven by a dual clock mechanism: the voltage clock (hyperpolarization-activated cyclic nucleotide-gated cation channel (*Hcn4*)) [5] and the calcium clock (ryanodine receptor (*Ryr2*), the sodium-calcium exchanger (*Ncx*) and the voltage-dependent calcium channel, T type, alpha 1G subunit (*Cacna1g*)) [6]. The slow conduction properties of nodal tissue are determined by the near absence of the cardiac sodium channel pore forming subunit Na_V_1.5 (encoded by *Scn5a*) and the preferential expression of low conductance gap junction proteins Cx30.2 and Cx45 (encoded by *Gjd3* and *Gja7*, respectively). On the contrary, fast conduction tissues, such as the pectinated atrial myocardium and VCS, are enriched in Na_V_1.5 and high conductance gap junction proteins Cx40 and Cx43 (encoded by *Gja5* and *Gja1*, respectively).

The nodal (slow) or fast conduction gene expression profiles are determined by the underlying transcription factor networks. One of the key transcription factors is the homeobox factor *Nkx2-5*, which when absent results in embryonic lethality due to arrested cardiac development. *Nkx2-5* is a critical driver of the fast conduction gene program and a potent inhibitor of the pacemaker program [7,8]. The ability of *Nkx2-5* to carry out these functional roles is dependent on its own expression level, as well as co-expression of other transcriptional regulators, such as the T-box factors TBX3 and TBX5. The repressive factor TBX3 turns off fast conduction gene programming (*Scn5a*, *Gja1*/Cx43, *Gja5*/Cx40), favoring a nodal phenotype, while the activator TBX5 promotes a fast conduction and pectinated/trabeculated myocardial (*Nppa*) phenotype. It should however be stated that TBX5 plays an important role in both nodal and fast conduction tissue development, and so, the functional role of TBX5 can be modified based on the tissue context. Paracrine and autocrine signals from endocardial, epicardial and myocardial cells play an important role in establishing tissue-specific, transcriptional hierarchies that ultimately determine the electrophysiological properties of CCS cell types. Herein, we will discuss these gene regulatory networks in their tissue-specific context.

## 2. The Sinus Node

**Figure 3:**
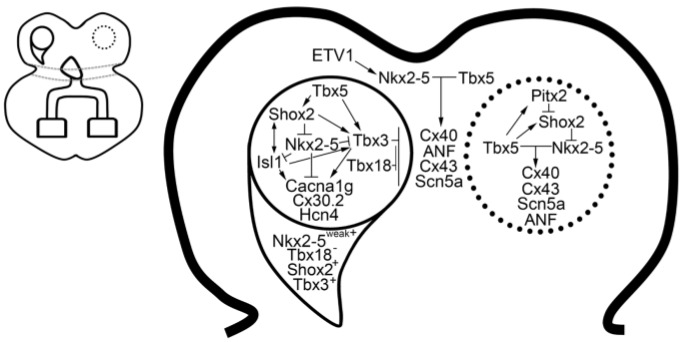
Sinus node gene regulatory network. The right-sided sinus node is partitioned into the head region (circle) and tail region (half crescent). The default left-sided sinus node gene regulatory network (dotted circle) is suppressed by Pitx2. Schematic overview of the cardiac conduction system, left panel; sinus node schematic, right panel (Reproduced with permission from [14]).

The mammalian sinus node is a comma-shaped structure divided into two main components: the head and tail regions [9] (Figure 3). The SN head and tail regions are located within the crista terminalis, which is the adult junction formed from the embryonic right venous valve, which is composed of sinus venosus and atrial tissues [10]. Sinus node dysfunction in mammals can manifest as sinus bradycardia, sinus exit block, sinus arrest or tachycardia-bradycardia syndrome. During development, a second wave of myocardial progenitor cells from the posterior second heart field contribute to the venous pole of the primary heart tube, contributing to the atria, sinus venosus (SV) and SN [11]. In the embryonic heart, SV myocardium is divided into the right and left sinus horns. The right sinus horn contributes to the SN, whereas the left sinus horn expresses *Pitx2c* that turns off the left-sided nodal gene program [12,13].

The critical gene networks that govern SN development are the T-box factors (*Tbx5*, *Tbx3* and *Tbx18*) and the homeodomain factors (*Shox2*, *Islet-1* (*Isl1*) and *Pitx2c*). TBX5 resides atop of this network driving the expression of *Shox2* and *Tbx3*. In the absence of *Tbx5*, mice die at embryonic Day 10.5 (E10.5) with severe hypoplasia of the inflow tract region [15]. *Tbx5* haploinsufficient mice exhibit sinus node dysfunction [16] and have reduced expression of *Shox2* and *Tbx3* in the inflow tract [17,18].

TBX3 is expressed in the SN, AVN and in the proximal HPS, where it represses chamber myocardial gene programming [19]. The SN is highly sensitive to *Tbx3* gene dosage, where graded loss of *Tbx3* is associated with ectopic upregulation of chamber-specific genes (*Gja1*/Cx43, *Gja5*/Cx40, *Nppa* and *Scn5a*) in the SN region [20,21]. In contrast, forced expression of *Tbx3* in the atrial myocardium results in downregulation of chamber genes and upregulation of nodal genes (*Hcn4*, *Gjd2*/Cx30.2 and *Cacna1g*) [21]. Expression of *Tbx3* in the SN is directly regulated by Baf250a, a component of the chromatin remodeling complex SWI/SNF [22]. Baf250a, TBX3 and the histone deacetylase 3 (HDAC3) then act coordinately to repress *Nkx2-5* expression in the SN [22].

*Tbx18* is expressed in the sinus horns during development. *Tbx18* expression is maintained in the sinus head until birth, whereas tail region expression is significantly downregulated during development [9]. *Tbx18* knockout (KO) results in a loss of the SN head, whereas the tail region is unaffected [9]. Despite loss of the head region, SN function remains intact, suggesting that the tail region is sufficient for normal pacemaker function. Ectopic expression of TBX18 in vivo is sufficient to convert working ventricular myocytes into SN-like cells based on morphology and function [23].

*Shox2* is essential for SN development, and deficiency of *Shox2* results in embryonic lethality due to SN hypoplasia and bradycardia [24]. SHOX2 represses expression of *Nkx2-5*, which is known to negatively regulate the expression of pacemaker genes (*Tbx3* and *Hcn4*) and to positively regulate the expression of chamber myocardial genes (*Nppa*, *Gja1*/Cx43 and *Gja5*/Cx40) [24,25]. Therefore, loss of *Shox2* results in ectopic upregulation of *Nkx2-5* and chamber myocardial genes in the SN [24]. Hypomorphism of *Nkx2-5* rescues the morphological and functional defects of the Shox2 KO mutant [7]. SHOX2 has been shown to directly regulate the expression of *Isl-1*, although an indirect mechanism whereby SHOX2 regulates *Isl1* expression via *Nkx2-5* has also been proposed [7,26]. An *Hcn4*-restricted knockout of *Isl-1* results in SN hypoplasia and sinus node dysfunction [27]. Similar to *Shox2* KO, *Hcn4*-restricted *Isl-1* KO SN exhibits reduced expression of pacemaker genes (*Tbx3, Hcn4, Hcn1* and *Cacna1g*) and ectopic expression of chamber myocardial genes (*Nppa*, *Gja1*/Cx43, *Gja5*/Cx40 and *Scn5a*) [28]. Overexpression of Isl-1 in *Shox-2* KO was able to rescue the SN phenotype in a zebrafish model [26].

During development, left-right asymmetry in the heart is regulated by the homeodomain transcription factor PITX2c, which is broadly expressed in left-sided cardiac structures, including the left SV myocardium, left atrium (LA) and pulmonary veins (PVs) [29,30]. *Pitx2c*-deficient mice exhibit right isomerism and the formation of a left SN, which shares an identical gene program as the right SN [13]. PITX2c negatively regulates *Shox2* expression through direct [31] and indirect [32] mechanisms in the developing left SVC and LA. *PITX2* has been identified as a key susceptibility locus for atrial fibrillation in genome-wide association studies (GWAS) [33]. Mice haploinsufficient for *Pitx2c* are predisposed to atrial fibrillation and express an ectopic SN gene program in the left SVC and posterior wall of the LA [31]. *Pitx2* expression is regulated by TBX5, and in turn, PITX2 antagonistically modulates the TBX5-dependent gene regulatory network, which includes *Scn5a* and *Gja1/*Cx43 [34]. Therefore, PITX2 negatively regulates a default gene regulatory network that drives right isomerism and nodal gene programming in the left heart.

## 3. The Atrioventricular Canal and Node

The mature AVN is a complex heterogeneous structure composed of nodal and transitional cells and remains the last point of communication between the atria and ventricles. The AVN is located within the triangle of Koch, which is bordered by the tendon of Todaro, antero-septal leaflet of the tricuspid valve and the os of the coronary sinus. Several properties of the AVN are essential for proper cardiac function: (1) the AVN functions as a subsidiary pacemaker if the SN fails; (2) slow conduction of the AVN provides adequate time for atrial contraction to fill the ventricles; and (3) decremental conduction of the AVN safeguards against rapid ventricular stimulation during supraventricular arrhythmias. Diseases of the AVN can manifest as variable degrees of AV block including complete heart block, as well as tachyarrhythmias such as AV nodal reentrant tachycardia.

**Figure 4:**
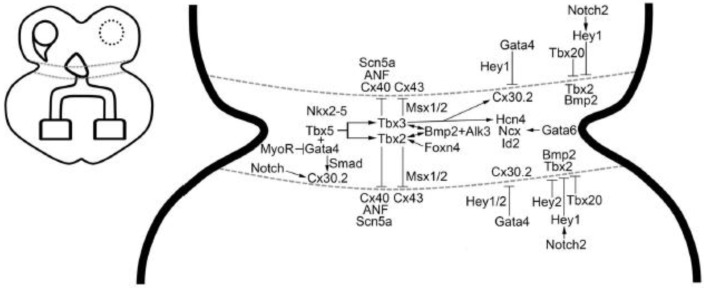
Atrioventricular canal/atrioventricular node gene regulatory network. Schematic overview of the cardiac conduction system, left panel; atrioventricular canal/node, right panel (reproduced with permission from [14]).

The AVN originates from precursor cells in the dorsal aspect of the AV canal (AVC) [35,36]. During development, the entire AVC functions as the primordial AVN and shares many of the gene regulatory networks that dictate its electrophysiological properties (Figure 4). In the adult mouse heart, the AVC myocardium gives rise to AV ring bundles that form figure-eight rings of nodal and transitional cells at the atrioventricular junction [4]. These AV ring bundles allow for a gradual electrophysiological transition from an atrial (fast conduction) gene program to a nodal (slow conduction) gene profile [4]. The caudal aspect of the nodal ring bundle makes direct contact with the compact AV node [4]. In the human heart, transitional cells are also present between the compact AV nodal cells and working atrial myocytes [37]. At the distal aspect of the AV node, the transition between the compact node and the penetrating His bundle is less histologically distinct. The definition of the His bundle, as originally defined by Sunao Tawara in 1906 [38], is the point at which the AV bundle penetrates the central fibrous body. The Tawara definition is functionally validated as atrial myocytes cannot directly activate the insulated His bundle apart from the AV node.

A multi-tiered transcriptional network maintains the AVC gene signature that maintains nodal properties, low proliferative capacity [2] and proper boundary formation with adjacent working myocytes. One of the best characterized gene regulatory networks involves BMP2, TBX2 and TBX3, all three of which are enriched in the AVC myocardium during development [39,40]. BMP2 maintains slow conduction in the AVC myocardium in two ways: (1) BMP2 contributes to AV cushion formation by directing endocardial epithelial-to-mesenchymal transition (EMT) and formation of cardiac jelly [39]. Endocardial cushion formation distances the endocardium, which secretes factors that enhance fast conduction gene programming, from the underlying AVC myocardium [41]. (2) BMP2 functions in a feed-forward loop with TBX2 and TBX3 to maintain a nodal gene program [39,40]. BMP2 has also been implicated in epicardial EMT [42], which contributes to the annulus fibrosus and electrical isolation of the atria from the ventricles.

Therefore, loss of *Bmp2* [39], BMP receptor (*Alk3*) [43,44] or *Tbx2/Tbx3* [40] is associated with developmental abnormalities of the AVC, manifesting as AV conduction abnormalities or defective annulus fibrosus formation, that can result in ventricular pre-excitation (i.e., Wolff-Parkinson White (WPW)). Loss of *Bmp2* results in downregulation of *Tbx2/Tbx3* and ectopic expression of chamber myocardial genes in the AVC [39,40]. AVC-specific KO of *Alk3* leads to defective annulus fibrosus formation, ventricular pre-excitation and structural and functional defects in the AVN [43,44]. Human correlates have been identified in familial forms of WPW syndrome that associate with *BMP2* [45] and *TBX3* [46].

The T-box repressors, TBX2 and TBX3, have redundant functions in the AVC, where they maintain a nodal gene signature [19,47,48]. However, *Tbx3* and *Tbx2* are not uniformly expressed in the heart, with *Tbx3* more highly expressed in the right AVC and *Tbx2* more highly expressed in the left AVC [35]. Proper *Tbx3* gene dosage is essential for establishing and maintaining the nodal gene program in the AVC/AVN. Graded loss of *Tbx3* in mouse models results in a dose-dependent perturbation of AV conduction and fetal demise [20]. In keeping with its role as a repressor, loss of *Tbx3* results in ectopic upregulation of chamber myocardial genes in the AVN [20,49]. Furthermore, conditional knockout of *Tbx3* at an adult time point produces AV block, indicating a role for TBX3 in the maintenance of AVN function postnatally [20].

*Tbx2* deficiency is embryonically lethal due to AVC differentiation defects and outflow tract septation abnormalities [50]. Given the higher levels of *Tbx2* on the left AVC, *Tbx2* KO displayed predominantly left-sided AVC defects, which include ectopic expression of chamber genes and disruption of the annulus fibrosus, producing left-sided accessory pathway formation and ventricular pre-excitation (WPW) [51]. *Tbx2* deficiency had minimal effect on the AVN, which resides within the triangle of Koch in the dorsal aspect of the interatrial septum where Tbx3 expression dominates [51].

Combined loss of *Tbx2* and *Tbx3* results in failure of AVC formation, as evidenced by a lack of AV constriction and AV cushion formation [40]. In addition to the ectopic upregulation of chamber genes, there was a significant downregulation of *Bmp2* levels [40]. On the other hand, forced expression of *Tbx2* or *Tbx3* in chamber myocardium downregulated chamber genes and upregulated *Bmp2* expression, which resulted in ectopic endocardial EMT and AV cushion formation [40]. In the AVC, TBX2 and TBX3 are known to interact with MSX1 and MSX2 to negatively regulate Cx43 expression [47]. These data reinforce the hypothesis of a feed forward loop between *Bmp2* and *Tbx2*/*Tbx3* that restricts nodal gene programming and AV cushion formation to the AVC [40].

Further refining the boundaries of the AVC are *Hey1* and *Hey2*, which are expressed in a complementary fashion to *Bmp2* [52]. *Hey1* is expressed in the atrium and ventricles, whereas *Hey2* is exclusively expressed in the ventricles. Both HEY1 and HEY2 suppress *Bmp2* expression, with the former being regulated by a Notch2-dependent mechanism [52]. Loss of *Hey2* in mouse and zebra fish results in expanded *Bmp2*- and *Bmp4*-expressing AVC regions, respectively [52]. Another mechanism for delimiting the AVC utilizes *Tbx20*, which is expressed in the atria and ventricles. TBX20 confines the expression of *Tbx2* in the AVC by inhibiting its promoter activity [53].

A unifying mechanism by which the AVC gene signature is established utilizes GATA-dependent regulatory switches within AVC enhancer regions [54]. Mutation of these GATA sites results in ectopic AVC enhancer gene expression in the chamber myocardium. Analysis of several AVC-enriched genes, such as *Bmp2*, *Tbx2*, *Tbx3*, *Msx2*, *Gjd3*/Cx30.2 and *Cacna1g*, showed enrichment of histone H3 lysine 27 (H3K27) acetylation marks, which is associated with active enhancers [54]. GATA4 was shown to act in conjunction with BMP2/SMAD signaling to recruit histone acetyl transferase to activate AVC gene transcription [54]. Conversely in the chamber myocardium, GATA4 recruits pan-cardiac histone deacetylases (HDACs) and HEY1/HEY2 resulting in H3K27 deacetylation and AVC gene repression [54].

*Tbx5* is expressed in the AVC and is essential for normal development of the AVN [17]. *Tbx5* haploinsufficient mice had underdeveloped AVC/AVN and displayed variable degrees of AV block [15,16]. TBX5 regulates the expression of GATA4, and together, TBX5 and GATA4 form a ternary complex that drives the expression of the low conductance gap junction protein *Gjd3*/Cx30.2 in the AVC [17,55]. *Gata4* haploinsufficient mice have structurally normal AVN, but have reduced expression of Cx30.2, resulting in more rapid conduction through the AVN [55]. The beta helix-loop-helix transcription factor MyoR inhibits GATA4 activation of the *Gjd3* minimal AVC enhancer [56]. TBX5 has also been shown to partner with the transcription factor Foxn4 to activate *tbx2b* promoter activity in zebra fish [57].

Gata6 is also an important regulator of AVN development [58]. Myocardial-specific ablation of the carboxyl zinc-finger domain of GATA6 demonstrated prolonged AV conduction due to a hypomorphic AVN, as well as cell cycling defects in the AV bundle [58].

The homeodomain factor *Nkx2-5* is an essential regulator of cardiomyocyte differentiation and impacts cardiac conduction system development at multiple levels. Loss of the *Nkx2-5* homolog, *tinman*, in fruit flies results in failure of cardiogenesis [59]. Mice deficient in *Nkx2-5* exhibit embryonic lethality at ~E9.5 due to failure to progress beyond partial looping morphogenesis with defects in AVC formation and trabecular development [60]. *Nkx2-5* haploinsufficient mice display hypoplasia of the AVN and VCS and demonstrate variable degrees of heart block that progresses with age [61]. *Nkx2-5* mutations in man are associated with non-syndromic congenital heart disease and AVN disease [62] due to fibrofatty replacement and myocyte dropout [63].

Notch signaling also plays an important role in AVN specification and annulus fibrosus maturation [64]. The dominant negative Notch mutant, DNMAML, exhibits reduced AVN volume due to a loss of Cx30.2-expressing cells, resulting in more rapid conduction through the AVN [64]. Constitutive Notch activation produced enlarged AV nodes and accessory pathway formation, resulting in ventricular pre-excitation [64]. Notch gain-of-function mice showed reduced levels of canonical Wnt signaling in the AVC [65]. Wnt signaling regulates the expression of *Bmp4* and *Tbx2b* in the AVC of zebra fish [66]. Loss of Wnt signaling in mice results in structural abnormalities of the tricuspid valve, right ventricle and loss of AVC myocardium [65], whereas ectopic Wnt signaling expanded the boundaries of the AVC and slow conduction gene programming in mice [65] and fish [66].

## 4. The Ventricular Conduction System

**Figure 5:**
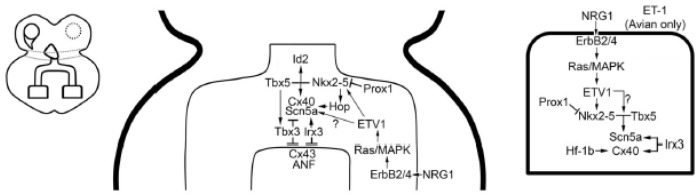
Ventricular conduction system gene regulatory network. Schematic overview of the cardiac conduction system, left panel; His bundle and bundle branch schematic, center panel; Purkinje cell schematic, right panel (Reproduced with permission from [14]).

During normal sinus rhythm, the ventricular myocardium is exclusively activated by the VCS. The VCS is composed of Purkinje cells, which have been implicated as the site of arrhythmia initiation in numerous inherited and acquired rhythm disorders that are associated with sudden cardiac death [67,68,69,70]. In addition, conduction disease in the VCS can manifest as complete heart block or as bundle branch block. Bundle branch block has been shown to increase morbidity and mortality in heart failure patients due to ventricular dyssynchrony [71]. Given the clinical importance of Purkinje cell biology in maintaining rhythm stability and optimal hemodynamics, understanding the molecular determinants of Purkinje cell specification and function has become essential. Significant advances have been made in our understanding of the gene regulatory networks that establish the VCS (Figure 5).

The VCS derives from ventricular trabecular myocardium during cardiac chamber formation. In the human heart, the proximal VCS (His bundle and bundle branches) forms from a GlN2^+^ trabecular myocardial pool that resides at the crest of interventricular septum and extends into the left and right ventricles [72]. This band of GlN2^+^ myocytes is directly continuous with the GlN2^+^ pool in the right aspect of the AV canal that contributes to the AVN within the lesser curvature. The distal Purkinje fiber network is added to the VCS at later developmental stages and postnatally after GlN2 expression has significantly declined. Similar to GlN2, TBX3 is expressed abundantly within the AVN and proximal VCS, but is either not present or expressed at significantly reduced levels in the distal Purkinje fiber network [19]. The differential expression of TBX3 drives a gradient of expression of *Gja5*/Cx40 in the developing VCS with the lowest levels in the His bundle and proximal bundle branches [49]. As development proceeds, the enrichment of NKX2-5, TBX5 and other transcription factors that enhance *Scn5a* and *Gja5*/Cx40 expression within the VCS counterbalance the repressive effects of TBX3.

The trabecular-derived VCS shares many of the fast conduction gene program seen in the pectinated atrial myocardium. The pectinated atrial myocardium and VCS are enriched in *Scn5a* [73] and *Gja5*/Cx40 [74], which defines these cell types throughout cardiac development. Both *SCN5A* [75] and *GJA5* [76] have been implicated in inherited forms of progressive cardiac conduction disease or Lev-Lenegre disease [77,78], which is characterized by fibrosclerotic degeneration of the His-Purkinje system. The trabecular myocardium further specifies into the VCS through a gene regulatory network that is dependent on overlapping expression of *Nkx2-5* [61], TBX5 [16,79] and TBX3 [49], in addition to tissue-specific expression of the ets transcription factor ETV1 [1] and the Iroquois homeobox 3 (IRX3). Gene dosage of *Nkx2-5* [61,80] and *Tbx5* [16] is critical for proper development of the conduction system, and reduced levels of either factor results in significant structural and functional abnormalities of the VCS. Combined haploinsufficiency of *Nkx2-5* and *Tbx5* results in specification failure of the proximal VCS, although AV conduction is maintained into the early post-natal period [79].

*Nkx2-5* haploinsufficiency is associated with conduction disease in the AVN and VCS resulting from hypoplasia of these tissues [61,63,81,82]. *Nkx2-5* mutant mouse models accurately phenocopy the progressive AV conduction disease seen in patients with *Nkx2-5* mutations [63]. There is a marked reduction in Purkinje fiber network cellularity despite the trabecular myocardium appearing normal during development [81]. These data suggest a failure of Purkinje cell recruitment or Purkinje cell loss in the postnatal period. The remaining Purkinje cells were normal in cell size and action potential characteristics, suggesting that the conduction abnormalities noted on electrocardiography were due to hypoplasia of the VCS [81].

*Nkx2-5* gene dosage is tightly regulated by upstream modulators. Prospero-related homeobox protein 1 (Prox1) functions in concert with HDAC3 to negatively regulate *Nkx2-5* expression [83]. Combined haploinsufficiency of *Nkx2-5* and *Prox1* rescues to a significant extent the structural and functional defects of the AVN and VCS in *Nkx2-5* heterozygous mice [83].

**Figure 6:**
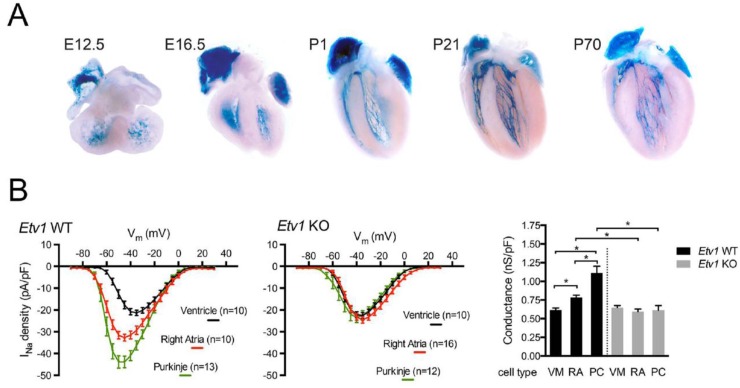
*Etv1*-nuclear LacZ expression during cardiac development. (**A**) *Etv1* is enriched in the pectinated atrial myocardium and in developing (trabecular myocytes) and mature Purkinje cells during cardiogenesis. Embryonic Days 12.5 and 16.5 correspond to E12.5 and E16.5; Postnatal Days 1, 21 and 70 correspond to P1, P21, and P70.; (**B**) Comparison of the sodium current-voltage (I–V) relationship between atrial, Purkinje and ventricular myocytes from *Etv1* WT and KO mice. Ventricular myocytes, VM; right atria, RA; Purkinje cell, PC. Data represent the mean ± S.E.M. * *p* < 0.05. *n* = 4 hearts per group (Panels A and B reproduced with permission from [1]).

The endocardium plays a critical role in establishing and maintaining the VCS. The endocardially secreted factor Neuregulin-1 (NRG1) has been shown to be sufficient to expand the expression of the Purkinje reporter gene (CCS-LacZ) throughout the embryonic heart [84]. We subsequently identified that NRG1 activates a fast conduction gene network in the pectinated atrial myocardium and VCS through the Ras-MAPK signaling pathway and the transcription factor ETV1 [1]. ETV1 is highly enriched in fast conduction tissues of the heart (Figure 6A). Loss of *Etv1* results in reduced levels of *Nkx2-5*, *Scn5a* and *Gja5*/Cx40, producing conduction abnormalities in the pectinated atrial myocardium and VCS, including bundle branch block [1]. *Nkx2-5* and *Scn5a* are reduced to ventricular levels in the pectinated atrial myocardium and VCS in *Etv1* KO hearts. Consequently, *Etv1* KO mice exhibit VCS hypoplasia mirroring the *Nkx2-5* heterozygous defect [61]. In addition, the normal biophysical differences in the sodium current between atrial, Purkinje and ventricular myocytes are lost in absence of *Etv1*, suggesting that ETV1 regulates a panel of genes that modulate the sodium current in the atria and VCS [1] (Figure 6B). Lastly, using phenome-wide association study (PheWAS) analysis, we identified a sequence variant in *ETV1* that associates with bundle branch block and heart block in humans [1].

*Tbx5* is also a key modulator of VCS specification and fast conduction gene programming. *Tbx5* haploinsufficient mice exhibit maturation defects of the His bundle and bundle branches, with some animals displaying an absence of the right bundle branch [16]. *Nkx2-5* and TBX5 cooperatively drive the expression of *Nppa*, *Gja5*/Cx40 and the transcription factor inhibitor of differentiation 2 (*Id2*) in the proximal VCS [79]. *Id2* KO mice display similar structural and functional defects in the VCS as seen in *Tbx5* heterozygous mice, suggesting they function in an overlapping gene network. Combined haploinsufficiency of *Tbx5* and *Id2* results in a specification defect of the proximal VCS, suggesting that *Nkx2-5*, TBX5 and Id2 function in a VCS transcriptome [79]. TBX5 is essential to maintain the fast conduction gene program in the mature VCS. A tamoxifen-inducible, VCS-specific *Tbx5* KO mouse displayed severe VCS conduction abnormalities, including Mobitz II second degree AV block and ventricular arrhythmias [85]. The expression of *Scn5a* and *Gja5* was significantly reduced in mutant VCS cells [86].

TBX3 is essential for normal development of the His bundle and proximal bundle branches [49]. Loss of *Tbx3* results in ectopic expression of Cx43, *Nppa*, *Tbx18* and *Tbx20* in the proximal VCS. His bundle and bundle branch cardiomyocytes deficient in Tbx3 failed to exit the cell cycle. These results support the hypothesis that TBX3 represses a working myocardial gene program in proximal VCS myocytes, while co-expression of NKX2-5, TBX5, and ETV1 in the VCS maintains a fast conduction gene program.

Genome-wide association studies (GWAS) have been used to identify novel genes that modulate ECG parameters, such as PR and QRS duration. These GWAS have identified several loci, including *TBX5*, *TBX3*, *NKX2-5*, *SCN5A* and *SCN10A* [87,88,89,90,91]. All of these genes except *SCN10A* had been well validated as modulators of VCS specification and fast conduction physiology. To elucidate the functional role of these *SCN10A* variants on cardiac conduction parameters, Moskowitz and colleagues examined the expression levels of *SCN10A* in cardiac tissues and found that *SCN10A* is at background levels in murine and human myocardial tissue [92]. Using a combination of ChIP-Seq and high-resolution 4C-seq analysis, a functional SNP rs6801957, which associates with QRS duration and Brugada syndrome [93], was shown to alter a TBX3/5 binding site [94] in a cardiac enhancer in the *SCN10A* locus that modulates *SCN5A* expression in mice and humans [92]. These data demonstrate the importance of GWAS in identifying novel genes and gene regulatory regions that underlie phenotypic variations on a population scale.

Cx40 is an essential component of the fast conduction gene program and maintains proper function of the VCS. Expression of Cx40 is first seen during atrial and ventricular chamber development within the pectinated atrial myocardium and trabecular myocardium. *Gja5*/Cx40 KO mice exhibit slowed conduction through the VCS and display bundle branch block [95,96]. Multiple redundant transcriptional networks reinforce Cx40 expression in fast conduction tissues. NRG1 activates ETV1 within pectinated atrial myocardium and trabecular/Purkinje myocytes to drive expression of *Nkx2-5* (1), which partners with TBX5 to activate Cx40 expression. Subsequently the transcription factors, Irx3, Hf-1b and Hop, further enhance the expression of Cx40 in the VCS.

IRX3 has been shown to have an important role in VCS specification and patterning. *Irx3* KO mice exhibit prolonged QRS duration and bundle branch block due to VCS hypoplasia and defects in right bundle formation [97]. IRX3 directly interacts with *Nkx2-5* and TBX5 and functions as a cofactor to drive Purkinje-specific gene programming. IRX3 directly downregulates Cx43 expression and upregulates Cx40 expression through an indirect mechanism [98]. Ambulatory monitoring of *Irx3* KO mice revealed spontaneous ventricular arrhythmias [99]. Screening of patients with idiopathic ventricular fibrillation identified two missense mutations in *IRX3*, both of which had reduced ability to increase *Gja5*/Cx40 or *Scn5a* expression in heterologous expression systems [99].

Hf-1b is a zinc-finger transcription factor that is expressed in the ventricular myocardium and VCS [100]. *Hf-1b* KO mice exhibit SND, AV block and spontaneous ventricular arrhythmias. Histological evaluation revealed a reduced number of Cx40+ distal Purkinje fibers and abnormal distribution of Cx40 within remaining cells, suggesting a trafficking defect. However, additional abnormalities were also present, including abnormal coronary artery structure and smaller apical myocardial cells that had reduced expression of Cx43 [101]. Therefore, it is unclear whether changes in Cx40 expression are cell autonomous or a consequence of the apical defects.

The homeodomain-only protein (HOP) is a unique member of the homeobox transcription factors that does not directly bind DNA. *Hop* KO mice have normal VCS structure, but exhibit conduction slowing in the VCS [102]. Cx40 levels were reduced in the atria and VCS in mutant hearts [102].

## 5. Conclusions

Knowledge of the gene regulatory networks that dictate the unique biophysical properties of the cardiac conduction system is expanding rapidly. The combination of genetic tools, such as GWAS, PheWAS, 4C-seq, RNA-seq and ChIP-seq, coupled with traditional murine and biochemical tools have accelerated the pace of discovery. The challenge for the future will be to understand how these gene regulatory networks function in a hetero-cellular context, where growth factor-dependent signal transduction gradients establish tissue-specific and region-specific expression of transcriptional networks.
